# Should I drink responsibly, safely or properly? Confusing messages about reducing alcohol-related harm

**DOI:** 10.1371/journal.pone.0184705

**Published:** 2017-09-21

**Authors:** Sandra C. Jones, Sabine Hall, Kypros Kypri

**Affiliations:** 1 Centre for Health and Social Research, Australian Catholic University, Melbourne, Victoria, Australia; 2 Centre for Clinical Epidemiology and Biostatistics, School of Medicine and Public Health, University of Newcastle, Callaghan, New South Wales, Australia; George Institute for Global Health, INDIA

## Abstract

‘Responsible drinking’ campaigns emerged in the early 1970s as a means of addressing hazardous drinking and its related consequences. While these were initially the product of public health agencies and health-related NGOs, they are increasingly being developed and disseminated by the alcohol industry. There is considerable debate as to whether industry-generated campaigns are designed to reduce hazardous drinking and related problems (as argued by their developers) or are designed to avoid government regulation or even to increase sales. The aim of the present study was to explore the way that recent industry-developed responsible drinking campaigns are perceived and interpreted by the general public. That is, do they promote low-risk drinking, promote risky drinking, or just muddy the waters. Two sub-studies were conducted. The first, a mall intercept study with 180 adults in two Australian shopping districts, explored participants’ understanding of slogans/taglines. The second, an online survey with 480 Australian adults, explored understandings and interpretations of television/online commercials. The results of the two studies revealed diversity in participants’ interpretation of the ‘responsible drinking’ advertisements. Terminology utilised in industry-developed advertisements was found to be ambiguous; for example, what age group was being referred to in the tagline ‘Kids and alcohol don’t mix’, and whether ‘Drink Properly’ meant not drinking to excess or drinking in a way that made you look more sophisticated. In Study Two, the government-developed campaign (‘Know when to say when’) was clearly interpreted as warning against risky consumption of alcohol; whereas the industry-developed campaigns (‘How to drink properly’, ‘Kids absorb your drinking’, ‘Friends are waiting’) were interpreted to have a range of different meanings, including some seemingly unrelated to alcohol. These findings are consistent with the literature evaluating anti-smoking campaigns developed by the tobacco industry, and previous research showing that industry communications serve to soften public opinion and create the impression of a ‘socially responsible’ industry but are likely to be less effective than initiatives focused on the availability and promotion of alcohol.

## Introduction

According to the Australian Bureau of Statistics, there were 9.7 litres of pure alcohol available for consumption per person in 2013–14 [1]. Major diseases and injuries attributable to alcohol consumption include alcohol use disorders, liver cirrhosis, cancers, suicide, violence, cardiovascular disease and foetal alcohol syndrome, while socioeconomic consequences include loss of income, unemployment, family problems, stigma and barriers to accessing health care [2].

As part of the strategy to reduce these harms, the Australian Government’s National Health and Medical Research Council (NHMRC) developed evidence-based guidelines which include drinking no more than two standard drinks (20g ethanol) on any day to reduce the lifetime risk and no more than four (40g ethanol) in a single session to reduce the short-term risk of harm from alcohol-related disease and injury [3].

At the same time, alcohol advertising is extensive, with marketing platforms including television, radio, YouTube, social networks, sporting and music events and branded merchandise [4]. The link between exposure to alcohol advertising and drinking initiation, levels of consumption and drinking patterns among young people has been established through longitudinal studies and systematic reviews [5–7]. Despite these links, the co-regulation and self-regulation of the alcohol industry ensures that their interest in marketing alcohol extensively continues to be served [8].

### Alcohol industry use of social aspects and public relations organisations ‘SAPROs’

The interests of the alcohol industry are represented by a range of organisations. First, there are the corporations themselves, many of which are transnational. Second, there are national and international organisations that represent the interests of the industry. The International Alliance for Responsible Drinking (IARD) is the merging of two alcohol industry organisations, the Global Alcohol Producers Group (GAPG) and the International Centre for Alcohol Policies (ICAP), with members from all sectors of the alcohol industry–beer, wine, and spirits. Australia has the Distilled Spirits Industry Council of Australia (DSICA), the Brewers Association of Australia & New Zealand and the Winemakers’ Federation of Australia (WFA) [9]. Similarly, the United States has the Distilled Spirits Council of the United States (DISCUS), the National Beer Wholesalers Association (NBWA), the Wine and Spirits Wholesalers of the United States (WSWA); and the United Kingdom has the Wine and Spirit Trade Association (WSTA), British Beer and Pub Association (BBPA) and many others.

Third, in many countries the industry has established organisations which they describe as ‘social aspects and public relations organisations’ (or SAPROs). Examples of SAPROs include the Foundation for Advancing Alcohol Responsibility (formerly known as the Century Council) in the United States, DrinkAware in the United Kingdom and DrinkWise in Australia. Depending on the eye of the beholder, these organisations are either funded by the industry to help reduce alcohol-related harm or to serve as a front for the industry to lobby for ineffective approaches and against effective countermeasures. Established in 2005, DrinkWise Australia was developed and funded by the alcohol industry, and industry representatives sit on its board; supplemented by $5 million from the Federal government in 2006. DrinkWise describes itself as an independent, not-for-profit organisation whose “primary focus is to help bring about a healthier and safer drinking culture in Australia;” and whose stated aims are to “promote a generational change in the way Australians consume alcohol” and to “increase the age that young Australians are introduced to alcohol” [10]. However public health advocates argue that SAPROs such as DrinkWise have been used to promote interventions that maintain profit, while diverting attention away from effective public health policies [11]. Further, that “Key strategies (of alcohol industry groups) include: industry-run education programs, focussing blame on individuals with a “problem”, promoting responsible drinking, denying any association between advertising and consumption, and focussing attention on minority groups” [12].

### ‘Responsible drinking’ campaigns

Responsible drinking campaigns emerged in the early 70s as a means of addressing risky drinking and related consequences [13]. They were underpinned by the philosophy that “there are two decisions a person can make about alcohol—either not to use it or to use it responsibly” [14]. Early examples include the Seattle Education Service District #121 ‘Here’s Looking at you’ campaign and the Cambridge-Somerville Program for Alcoholism Rehabilitation ‘Decisions About Drinking’ campaign [15].

Increasingly, alcohol industry SAPROs are developing and disseminating responsible drinking campaigns. While SAPROs also assert that their aim is to reduce risky drinking and related problems, public health experts argue that their messages are too ambiguous to be effective and are really designed to serve a public relations function, to increase sales and to minimise regulation [16].

A recent example is Drinkwise’s ‘How to Drink Properly*’* campaign which was launched in 2014 and purportedly aimed to influence young adults to drink responsibility [10]. The animated campaign was promoted primarily through social media, and used a handsome, smooth character (described as being like Don Draper from Mad Men, or James Bond) to encourage young people to moderate the intensity and frequency of binge drinking episodes through speaking “in their own language”. For example, their website introduction says: “here you’ll find everything you need to know about keeping your s*** together when you drink. Peruse our videos, or click through to our classy as f*ck social pages to discover the difference between drinking, and drinking properly.” However, the campaign received extensive criticism from public health experts due to its glamorisation of drinking as sophisticated and stylish and lack of clarity around what constitutes drinking too much [12]. A qualitative study conducted with 48 18-21-year-old drinkers found that participants liked the ‘How to Drink Properly’ YouTube advertisement and it appeared to reinforce existing social norms relating to risky alcohol consumption at social events [17].

### Aim of the study

The aim of the present study was to explore the way that recent SAPRO-developed responsible drinking campaigns are perceived and interpreted by the general public. Specifically, do members of the public interpret SAPRO-developed advertisements:

(a) as promoting low-risk drinking (as claimed by the industry); [10] or
(b) as promoting risky drinking (as claimed by some public health advocates [18]; or
(c) as ambiguous (as suggested by others) [19].

Further, we sought to explore:

(d) how the clarity of these messages compared to a government-developed campaign.

Study One (an intercept survey) enabled us to explore consumer understandings of slogans/taglines; and Study Two (an online survey) to explore understandings and interpretations of television/online commercials.

## Methods

We undertook two studies using mixed methods: face-to-face (Study One) and web-based (Study Two) surveys involving the selection of response options or free-text/open-ended responses with qualitative analysis.

### Study One: Intercept survey

#### Participants and recruitment

Participants aged 16 years and over were recruited using mall intercepts in the Central Business District (CBD) shopping areas in Melbourne, Victoria (n = 90); and Newcastle, New South Wales (n = 90). The mall intercept survey is a widely used method of data collection in marketing research [20]. Quotas were established for gender (45 male and 45 females per location) and age (approximately 30 respondents per location, aged 16–25, 26–45, and 46 years and over). Respondents were provided with a small chocolate bar as a token of thanks for participating in the research. To ensure anonymity, participant contact forms (completed by those who wished to receive a copy of the research results) were collected separately.

#### Instrument

Respondents were shown six different responsible drinking slogans/taglines in random order:

1. ‘Drink Smart’ [used by a range of organisations as diverse as Beam Suntory (one of the world's leading beverage alcohol producers) and the NSW Department of Education and Training];
2. ‘Know when to say when’ [from a NSW Government education initiative];
3. ‘You won’t miss a moment if you DrinkWise’ [DrinkWise];
4. ‘Drinking: Do it properly’ [DrinkWise];
5. ‘Kids absorb your drinking’ [DrinkWise]; and
6. ‘Kids and alcohol don’t mix’ [DrinkWise].

For each slogan/tagline, respondents were asked an open-ended question: “What does the following statement mean to you?”

They were then asked to identify the age group(s) referred to in the message ‘Kids and alcohol don’t mix’ [response options: children aged 0–6 years, children aged 7–12 years, children aged 13–15 years, and children aged 16–17 years]. Given the debate regarding the purpose of the Drinkwise ad ‘How to drink properly’ that was occurring at the time [17], participants were shown a still image of the ad and they were asked what the word ‘properly’ meant to them [response options: staying sober, looking cool when you drink, knowing how to handle your alcohol, drinking the right kind of alcohol, knowing your limits]. Both questions allowed for multiple responses and respondents were asked to select all that applied. Finally, they answered a series of demographic questions.

The survey instrument was pre-tested using one-on-one cognitive interviews with a convenience sample of 6 men and 13 women, 18 years of age and older, recruited from the researchers’ networks via email or phone [21]. This allowed the researchers to identify any clarity or logic issues and measure administration times. The interviewer asked additional questions to explore the appropriateness and comprehension of the wording for each item. The average time to complete the survey was 13 minutes. Minor revisions were made prior to study commencement; such as formatting, clarification of instructions, and reducing the number of response categories for some demographic questions.

### Study Two: Online survey

#### Participants and recruitment

Participants were recruited using a combination of advertising on social networking sites (n = 177) and a commercial recruitment agency to ensure our age and gender quotas were met (n = 310). A total of 487 people completed the online survey; seven participants were excluded because their responses were incomplete or clearly vexatious, leaving a sample of 480 for analysis.

#### Instrument

Survey Monkey 2013 was used as the survey platform. The online survey was pre-tested with the same convenience sample that pre-tested the intercept survey (6 men and 13 women aged 18 years and older). The average time to complete the online survey was 15 minutes. Minor revisions were made following pre-testing.

Demographic questions were asked immediately after completing the consent process, to enable the screening out of ineligible or over-quota respondents. Four video (TV or online) advertisements were then shown in random order (see Table 1). Following each ad, participants were asked an open-ended question: “What do you think is the main message of this ad?”. They were then asked who they believed to be the target audience [teenagers aged 12–17 years, young adults aged 18–25 years, adults aged 25 years and over, parents–select all that apply]; whether the ad was relevant to them; and whether they believed it would cause them to change their behaviour in any way. Respondents were also asked what each of the four taglines meant to them. Finally, they were directed to a separate site where they could provide their email address for entry into a random draw to win an AU$100 iTunes voucher.

**Table 1:** Description of advertisements shown to respondents (Study Two).

Advertiser	Tagline	Description of ad
Drinkwise Australia	‘How to drink properly’	Black and white ad featuring an animated James Bond-like character who tells viewers to avoid drinking “like an amateur” or risk ending up too drunk.
Drinkwise Australia	‘Kids absorb your drinking’	Ad shows a boy getting a beer out of the fridge for his dad, highlighting the relationship between the way parents drink and how their children grow up to drink.
Budweiser	‘Friends are waiting’	Shows the friendship a man shares with his dog. Things take a twist, when one night the dog’s owner fails to return home after a night of drinking with his friends. The ad tells viewers to “make a plan to make it home” because “your friends are counting on you”.
NSW Health	‘Know when to say when’	Ad shows the spectrum of consequences from drinking, not only for the drinker but also for people they come in contact with.

#### Data analysis

Descriptive analyses were computed for all demographic variables for Study One and Study Two participants.

For both Study One and Two, categorical data were entered into the Statistical Package for the Social Sciences (SPSS 22.0) and descriptive analyses were conducted.

The deductive coding of open-ended responses involved an iterative process whereby two independent coders (SJ and SH) reviewed the responses to Survey One and developed a list of codes; and then coded all responses against these codes. Initial poor levels of agreement indicated a need to further refine the coding guide. Thus two separate coders (JL and KK) reviewed a subset of the responses (those on which the initial coders differed) and coded them independently. Following discussion between the four coders the coding guide was revised with clear explanation of the meaning and inclusions of each code. The lead author (SJ) then re-trained one of the initial coders (SH) and an additional coder (JT) who independently coded all of the open-ended responses. The inter-rater agreement across the two reviewers was 99.4% for the intercept survey and 99.7% for the online survey. Discrepancies were resolved through group discussion with the lead author as arbiter.

## Results

### Study One: Intercept survey

Consistent with the quotas established, 90 respondents were recruited in each of Melbourne and Newcastle (45 men and 45 women in each location). Approximately one-third were aged 16–25 years (n = 58), 26–45 years (n = 62) and 46 years and over (n = 60). Additional demographic information on the sample is provided in Table 2.

**Table 2:** Demographic details of Study One and Study Two participants.

	Study One			Study Two		
	Male N = 90	Female N = 90	All N = 180	Male N = 235	Female N = 245	All N = 480
**Age**						
16–25 years	32%	32%	32%	32%	27%	29%
26–45 years	36%	33%	34%	35%	31%	33%
46+ years	32%	34%	33%	33%	42%	38%
**Country of birth**						
Australia	64%	69%	67%	74%	80%	77%
Other	36%	31%	33%	26%	20%	23%
**Language spoken at home**						
English only	71%	81%	76%	82%	93%	87%
Other	29%	19%	24%	18%	7%	13%
**Religion**						
Anglican	11%	10%	11%	9%	18%	14%
Catholic	17%	17%	17%	22%	26%	24%
No religion	48%	47%	47%	46%	39%	43%
Other	24%	26%	25%	24%	16%	20%
**Education**						
Primary school—Year 12	26%	27%	26%	36%	36%	36%
Certificate, trade, diploma	32%	23%	28%	26%	29%	28%
Bachelor degree–Postgrad	42%	50%	46%	38%	35%	36%
**Marital status**						
Married/ de facto	42%	52%	47%	48%	53%	50%
Divorced, separated, widowed	11%	11%	11%	10%	14%	12%
Never married/ single	47%	37%	42%	42%	33%	38%
**Income per annum**						
Under $31,200	43%	42%	43%	31%	38%	35%
$31,200–$77,999	24%	29%	27%	37%	27%	32%
$78,000 or more	32%	29%	31%	32%	35%	33%
**Indigenous status**						
Aboriginal/Torres Strait Islander	7%	2%	4%	2%	2%	2%

#### Perceived tagline meanings–targeting adult drinking

Across the four taglines targeting adult drinking (‘Drink Smart’; ‘Know when to say when’; ‘You won’t miss a moment if you DrinkWise’ ‘Drinking: Do it properly’) 17 unique interpretations were coded. This excludes those who simply stated that they agreed or disagreed with the message (between 11 and 22 across the four ads), gave a response that was too vague to code (n = 25 to 49), or did not provide a response (n = 6 to 10). These 17 codes were reduced to 10 over-arching codes (see S1 Appendix for codes, sub-codes and their definitions).

A wide range of meanings were proposed for the tagline *‘*Drink Smart*’*, with the most common being drinking in moderation (n = 44, 24%), thinking about what you are drinking (n = 42, 23%), knowing your limits (n = 30, 17%) and not drinking to drunkenness (n = 30, 17%). About half of the respondents (n = 108) identified that *‘*Know when to say when*’* was about knowing your limits and not drinking beyond those limits; this response was more common among females (66%) than males (54%). No other interpretation was identified by more than 6% of respondents.

The most common interpretation of ‘You won’t miss a moment if you DrinkWise’ was avoiding drunkenness (n = 63, 35%). It is noteworthy that 20.6% (n = 37) specifically identified the message as being able to remember things after a night of drinking/not having blackouts; this response was more common among females (26%) than males (16%). Other messages identified were drinking in moderation (n = 28, 16%) and still being able to have fun without excessive drinking (n = 26, 14%).

The most common interpretation of ‘How to drink properly’ was drinking in moderation (n = 60, 33%). Other interpretations identified by more than 20% of respondents were avoiding drunkenness (n = 22, 12%) and thinking about what you are drinking (n = 23, 13%). Fifteen respondents (8%) interpreted this message to be a call to drink more rather than less alcohol.

Given the debate referred to above, it is important to understand how respondents interpreted the term “properly”. Of the seven options presented to respondents (see Table 3), the most common interpretation of the slogan ‘drink properly’ was ‘knowing your limits’ (52%), followed by ‘knowing how to handle your alcohol’ (39%). Further, 24% stated that drinking properly meant ‘looking cool when you drink’ and 21% ‘drinking the right kind of alcohol’; whereas only 16% agreed that it also meant staying sober. There were no significant differences in responses by age or sex, but those with a diploma or degree were more likely than those with high school/trade level qualifications to interpret the slogan to mean: looking cool when you drink (32% vs 13%, **χ^2^ =** 9.27, p = 0.002); drinking the right kind of alcohol (28% vs 10%, **χ^2^ =** 8.62, p = 0.003); and knowing how to mix a drink (19% vs 7%, **χ^2^ =** 5.46, p = 0.02).

**Table 3:** Perceived meaning of ‘drink properly’ (Study One, n = 180).

Perceived meaning	
Knowing your limits	52%
Knowing how to handle your alcohol	39%
Looking cool when you drink	24%
Drinking the right kind of alcohol	21%
Staying sober	16%
Knowing how to mix a drink	14%
"Other"	16%

#### Perceived tagline meanings–targeting adults’ influences on children’s drinking

Eleven unique interpretations were coded across the four taglines targeting parents and other adults’ influence on children’s drinking; excluding those who simply stated that they disagreed with the message in either of the two ads (8 and 3 respectively), gave a response that was too vague to code (14 and 16 respectively), reported that they did not know what the message was (5 and 2 respectively), or did not provide a response (6 and 2 respectively). These 11 codes were reduced to seven over-arching codes (see S2 Appendix for codes, sub-codes and their definitions). Note that simply agreeing was kept as a code for these ads due to the high number of responses in this category for the final ad.

The majority of respondents (n = 165, 98%) thought that the tagline ‘Kids absorb your drinking’ meant that children imitate their parents’ behaviour, with 22% (n = 39) making a clear connection to alcohol (e.g. ‘kids emulate your drinking habits’); this latter interpretation was considerably more common among female (28%) than male (16%) respondents. No other interpretation was provided by more than 8% of respondents.

Almost half (n = 80) of the respondents indicated that ‘Kids and alcohol don't mix’ meant that children should not drink, or be provided with, alcohol. The next most common response (n = 45, 25.0%) was a statement of agreement with the message, although it is unclear what these respondents perceived that message to be. Approximately 12% perceived the message to be that they should drink responsibly in front of children (n = 22) and a similar number that they shouldn’t drink in front of children (n = 20).

Given that almost half of the respondents interpreted this last slogan to mean that they should not provide children with alcohol, it was important to know what they believed the word ‘children’ referred to. In response to that question, only 54% agreed that ‘kids’ in ‘kids and alcohol don’t mix’ also included 16–17 year olds; whereas 81% believed it also included 13–15 year olds and 75% that it also included 7–12 year olds.

### Study Two: Online survey

Consistent with the quotas established, approximately one-third of the 480 respondents were from Victoria (n = 156), New South Wales (n = 177) and the rest of Australia (n = 147). Approximately half were male (49%, n = 235), with a fairly even split across the three age groups of 16–25 years (n = 140), 26–45 years (n = 159) and 46 years and over (n = 181). Additional demographic information on the sample is provided in Table 2.

#### Perceived tagline meanings–targeting adult drinking

Twenty-two unique interpretations were coded across the three taglines; again this excludes those who simply stated that they agreed or disagreed with the message (between 3 and 6 across the three ads), gave a response that was too vague to code (n = 1 to 7), or did not provide a response (n = 9 to 16). These 22 codes were reduced to 11 over-arching codes (8 from Study One, and three that were specific to ads in Study Two, see S3 Appendix).

The predominant interpretation of the ‘Know when to say when’ advertisement was knowing your limits (n = 247, 52%). This was more common among females (56% vs 47%, χ^2^ = 3.99, p = 0.05, those aged 25 or less (64% vs 46%, χ^2^ = 11.61, p < 0.001) and those without a tertiary education (58% vs 45%, χ^2^ = 7.99, p = 0.005). Other common interpretations were not becoming drunk (n = 100, 21%), avoiding negative outcomes (n = 89, 18%) and drinking in moderation (n = 62, 13%).

Two interpretations were prominent in response to the ‘How to drink properly’ advertisement. The most common of these was knowing your limits (n = 222, 46%), including references to previous experience and knowing how much alcohol you can handle (Table 4). This was more common among women (51% vs 41%, χ^2^ = 4.99, p = 0.03), those aged less than 25 (58% vs 41%, χ^2^ = 10.71, p = 0.001), and those without a tertiary education (52% vs 41%, χ^2^ = 5.73, p = 0.02). Other commonly noted interpretations were drinking in moderation (n = 144, 30%), not becoming drunk (n = 63, 13%), and avoiding negative outcomes (n = 33, 7%)–both of the latter showed a particular emphasis on not embarrassing yourself. Nineteen respondents commented that the main message was about being sophisticated and 16 that it was to drink more.

**Table 4:** Perceived main messages (grouped) for adult-targeted ads (Study Two, n = 480).

Code	How to drink properly	Friends are waiting	Know when to say when
Drink in moderation	30%	18%	13%
Don’t get drunk	13%	<1%	21%
Think/drink smart	3%	17%	3%
Know when to stop	46%	1%	52%
Abstain	1%	<1%	<1%
Don’t drink and drive	0%	36%	<1%
Drink more	3%	1%	<1%
Think about outcomes	7%	10%	19%
Advertisement	0%	4%	1%
Be classy	4%	0%	0%
Friends/pets are waiting	0%	28%	0%

The predominant interpretation of the ‘Friends are waiting’ advertisement was don't drink and drive (n = 173, 36%). This was followed by friendship (n = 133, 28%), with half of these (n = 66, 50%) mentioning alcohol (e.g., ‘your friend the dog is worth more than drinking’) and the remainder not specifically referring to alcohol (e.g., ‘dogs are a man’s best friend’). Women were more likely than men to note friendship as the message (32% vs 23%, χ^2^ = 4.72, p = 0.03). A smaller proportion mentioned drinking in moderation (n = 88, 18%); thinking about what you are drinking (n = 82, 17%); and avoiding negative outcomes, with a particular focus on harm to others (n = 49, 10%). Those aged 25 or less were more likely to note the message as thinking about what you are drinking (23% vs 15%, χ^2^ = 4.65, p = 0.03%) and avoiding negative outcomes (21% vs 6%, χ^2^ = 23.80, p<0.001), and less likely to perceive the message as to drink in moderation (11% vs 21%, χ^2^ = 6.29, p = 0.01). Those with as tertiary education were more likely to perceive the message as drink in moderation (22% vs 15%, χ^2^ = 3.90, p = 0.05) and less likely to see it as avoiding negative outcomes (7% vs 14%, χ^2^ = 6.26, p = 0.01).

#### Perceived tagline meanings–targeting adults’ influences on children’s drinking

Nine unique interpretations were coded for the tagline ‘targeting parents and other adults’ influence on children’s drinking’; excluding those who simply stated that they disagreed with the message (n = 2), gave a response that was too vague to code (n = 25), or did not provide a response (n = 12). These nine codes were reduced to seven over-arching codes (see S4 Appendix). The most common response was that the tagline ‘Kids absorb your drinking’ meant that children imitate their parents’ behaviour, (n = 369, 77%), with 35% (n = 167) making a clear connection to alcohol (e.g. ‘kids emulate your drinking habits’). Ten percent (n = 48) perceived the message to be that you should drink responsibly in front of children and 8% (n = 39) that kids shouldn't drink. No other interpretation was identified by more than 8% of respondents. However, it is noteworthy that nine respondents stated that the ad’s message was to increase viewers’ alcohol consumption.

#### Perceived target groups

The primary perceived target group for the ‘How to drink properly’ and ‘Friends are waiting’ campaigns was young adults aged 18–25 years; whereas for the ‘Know when to say when’ campaign it was adults aged 25 years and over. Parents were seen as the main target audience for ‘Kids absorb your drinking’.

#### Perceived relevance

The advertisement participants were most likely to report was relevant to them was the US ‘Friends are waiting’ (42%), followed by ‘Know when to say when’ (37%), ‘How to drink properly’ (35%) and ‘Kids absorb your drinking’ (33%). Conversely, the ad perceived as most likely to change their behaviour was ‘Kids absorb your drinking’ (40%) and the ad least likely to impact on their drinking was ‘How to drink properly’ (23%). The only gender difference was that female participants were more likely to perceive ‘Friends are waiting’ as relevant to them (54% vs 46%, **χ^2^** = 4.061, p = 0.04).

## Discussion

The results revealed diversity in participants’ interpretations of the four advertisements, particularly in relation to taglines found to be ambiguous. For example, in Study One only slightly more than half of respondents agreed that ‘kids’ in ‘kids and alcohol don’t mix’ included 16–17 year olds, whereas the legal alcohol purchase age in Australia is 18 years and national guidelines clearly recommend that not drinking is the safest option for those aged under 18 years [3].

In the same study, ‘Drink Properly’ was commonly interpreted as not drinking to excess but one in four viewers thought it meant “look cool when you drink”, one in five that one should “drink the right kind of alcohol”, and one in seven that one should know “how to mix a drink”. There were also small proportions of viewers who thought the message was that they should drink more. It was interesting to note that those with tertiary qualifications were more likely to interpret the slogan to mean looking cool when you drink, drinking the right kind of alcohol, and knowing how to mix a drink–perhaps suggesting that the nuanced message was more evident, or the ‘sophisticated’ character more appealing, to this group. The results suggest that the ads succeed in a balancing act of promoting an ostensibly ‘responsible’ message while conveying the view that there is a cachet to be gained from drinking, and even from drinking a lot, if it’s done ‘properly’.

This is consistent with the literature evaluating anti-smoking campaigns developed by the tobacco industry [22–23]. In the alcohol context, Miller Brewing Company’s teen alcohol prevention booklet ‘Let’s Talk Over a Beer’ has been described as sending “a clear message that drinking beer…is an adult activity” [24]; the same strategy used by Philip Morris to ‘discourage’ youth tobacco smoking which was shown to make smoking more appealing to adolescents [25].

In Study Two, the government-developed campaign (know when to say when) was clearly interpreted as warning against harmful consumption of alcohol. However, both of the SAPRO-developed and industry-developed adult-targeted campaigns (Drinkwise, Australia and Budweiser, US respectively) were interpreted to have a range of different meanings–including some seemingly unrelated to alcohol (e.g., the focus on pets in response to the Budweiser commercial) and some seemingly encouraging or glamourising drinking (e.g., ‘be classy’ in response to the Drinkwise commercial).

The findings are consistent with previous research showing that industry communications about their products are self-serving, sometimes in ways that are not immediately obvious [12]. First, the available literature indicates industry-funded educational campaigns serve a PR function, leading to positive views of that industry as taking responsibility for reducing any potential harms associated with the product [26–27]. Second, it has been argued that a function of industry-driven initiatives, such as responsible drinking messages, is to pre-empt and thus avoid government regulation [28].

There were noteworthy differences in interpretation by level of educational attainment of the respondents. For both the ‘Know when to say when’ and ‘How to drink properly’ advertisements, those without a tertiary education were more likely to interpret the message as knowing your limits. This refers to a subjective assessment of a safe or appropriate level of alcohol consumption based on previous experience and knowing how much alcohol you can ‘handle’, rather than an objective assessment of a quantity of alcohol based on medical or health guidelines. Thus, it may be particularly important to develop messages for this group that communicate the unreliability of this assessment for avoiding short- and long-term harms from alcohol consumption. Conversely, those with a tertiary education were more likely to interpret the ‘drink properly’ slogan as a call to appear more sophisticated when drinking, for example to drink the right kind of alcohol and know how to mix a drink. Campaigns targeting this group may need to focus on countering perceptions of drinking as glamorous and indicative of social success or status.

### Limitations

Limitations of the study include the reliance on participant introspection and attribution as a basis for inference as to the meanings of the advertisements. However, we would argue that it is viewers’ interpretations of these meanings that are of primary importance, rather than the stated intentions of the advertiser. While a viewer’s impressions of advertisements are, by definition, subjective, it is possible that their behaviour, e.g., how they drink, is weakly correlated with the verbal or web-based responses given in a survey. A research context provides artificial conditions for the viewing of advertisements, and it is uncertain how this affects inferences. It may be expected that participants answer questions in ways they think might be expected from a university researcher, a phenomenon referred to as ‘research participation effects’ [29]. However, experimental evidence suggests that such reactivity would have been less in the web survey format of Study Two than in the face-to-face format of Study One [30]. Another limitation of the study is that we did not collect data from the participants on (the frequency of) their previous exposure to the advertisements and/or taglines. However, based on the high-profile nature of the DrinkWise campaign, its extensive presence on social media, and the associated news and trade press coverage our respondents are more likely to have been exposed to the SAPRO campaign than the government campaign. Further, the government campaign ran in only one of the two states in which we conducted the study. If, as a reviewer suggested, repeated exposure is likely to lead to increased clarity, this strengthens our argument that respondents’ inconsistent interpretations of the DrinkWise campaign reflect its ambiguity.

Strengths of the research include the use of two sampling approaches (street intercept and survey panel), and survey modalities (face-to-face and web), and different locations (Melbourne and Newcastle), suggesting that the findings, which were similar across the studies, are robust.

## Conclusion

The evidence for reducing the population’s exposure to the marketing activities of corporations that sell dangerous products such as alcohol is clear. However, despite substantial levels of public support for greater restrictions, successive governments in Australia–as in many other countries–have been reluctant to do so. The challenge is in securing changes in policy in the face of strong industry resistance to regulation, and skilled lobbying for the status quo or relaxation of existing restrictions.

We found that participants clearly understood the message of the government campaign as a warning against harmful drinking, but many found the SAPRO-developed campaigns to be ambiguous. This study adds to a small but consistent body of research in the Australian context that the activities of SAPROs, and other alcohol industry initiatives, serve to soften public opinion and create the impression of a ‘socially responsible’ industry while being ineffective in moderating consumption. There would be value in investigating policy makers’ perceptions of such initiatives and the role of SAPROs and industry lobbying in avoiding evidence-based regulatory changes to reduce alcohol-related harms.

## Supporting information

S1 Appendix Codes and sub-codes for adult-targeted ads (Study One). (DOCX)

S2 Appendix Codes and sub-codes for parent-targeted ads (Study One). (DOCX)

S3 Appendix Codes and sub-codes for adult-targeted ads (Study Two). (DOCX)

S4 Appendix Codes and sub-codes for parent-targeted ads (Study Two). (DOCX)

S5 Appendix Paper-based survey for Study One. (DOCX)

S6 Appendix Online-based survey for Study Two. (RTF)

S1 Dataset Minimal dataset for Study One. (XLSX)

S2 Dataset Minimal dataset for Study Two. (XLS)
