# Supplementary material for: Should I drink responsibly, safely or properly? Confusing messages about reducing alcohol-related harm
Source: PLoS One. 2017 Sep 21;12(9):e0184705. doi: 10.1371/journal.pone.0184705 (PMC5608266; doi:10.1371/journal.pone.0184705)
Supplement: S1 Appendix — (DOCX) [file pone.0184705.s001.docx]

**S1 Appendix. Codes and sub-codes for adult-targeted ads (Study One)**

| **Code** | **Sub-code** | **Example responses** |
| --- | --- | --- |
| Moderation | Responsible | Drink responsibly/be responsible |
|  | Moderation | Drink in moderation/drink moderately |
|  | Properly | Drink properly/drink proper |
| Drunkenness | Drunk | Don't get drunk/don't binge drink |
|  | Excess | Don't drink too much/don't drink to excess/in excess/excessively |
|  | Blackout | Still be able to remember things/don't have blackouts/don't forget night |
| Fun | Fun – abstain | Its ok not to drink/can still have fun if you don't drink |
|  | Fun – less | Its ok to drink less/can still have fun if you drink less |
| Think | Think/consider | Think about what you are drinking/consider what you are drinking/be aware of what you are drinking |
|  | Sensible/safe | Be sensible/make safe choices/have a plan/control the amount you drink |
|  | Minimisation | Pace your consumption/pacing/drink less/drink water in between |
| Know | Know your limits | Know your limits/know what you drink/know when to stop |
| Abstain | Abstain | Don't drink alcohol/don't drink any |
| Drive | Drink-driving | Don't drink and drive |
| Guidelines* | NHMRC guidelines | Drink within the guidelines/drink within the rules (includes those who state number of standard drinks) |
| Pressure* | Peer pressure | Resist peer pressure/don't give in to peer pressure |
| Increase | Drink more | Drink hard/drink more |

* Code not evident in Study Two
