# Supplementary material for: Should I drink responsibly, safely or properly? Confusing messages about reducing alcohol-related harm
Source: PLoS One. 2017 Sep 21;12(9):e0184705. doi: 10.1371/journal.pone.0184705 (PMC5608266; doi:10.1371/journal.pone.0184705)
Supplement: S2 Appendix — (DOCX) [file pone.0184705.s002.docx]

**S2 Appendix. Codes and sub-codes for parent-targeted ads (Study One)**

| **Code** | **Sub-code** | **Example responses** |
| --- | --- | --- |
| Modelling | Follow you | Kids learn by listening and watching/monkey see monkey do (no direct reference to alcohol) |
|  | Follow drinking | Kids follow/learn your drinking habits |
|  | Model | Be a role model/good role model |
| Pregnancy* | N/A | Don't drink when you are pregnant/don't drink when you are breastfeeding |
| Don't drink | Don't drink – children | Don't drink in front of children/don't drink when children are present |
| Responsible | Drink responsibly – children | Drink responsibly around children/don't drink excessively in front of children |
| Kids | No drinking | Kids shouldn't drink |
|  | Harm | Damage brain/damage development (reference to kids) |
|  | Supply | Don't give kids alcohol |
| Teach* | Teach drinking | Only give kids small amounts of alcohol/only with parents/teach kids to drink responsibly |
| Agree | Agree/like | Yes/that's correct/good slogan |

* Code not evident in Study Two
