# Supplementary material for: Should I drink responsibly, safely or properly? Confusing messages about reducing alcohol-related harm
Source: PLoS One. 2017 Sep 21;12(9):e0184705. doi: 10.1371/journal.pone.0184705 (PMC5608266; doi:10.1371/journal.pone.0184705)
Supplement: S3 Appendix — (DOCX) [file pone.0184705.s003.docx]

**S3 Appendix. Codes and sub-codes for adult-targeted ads (Study Two)**

| **Code** | **Sub-code** | **Example responses** |
| --- | --- | --- |
| Moderation | Responsible | Drink responsibly/be responsible |
|  | Moderation | Drink in moderation/drink moderately |
|  | Properly | Drink properly/drink proper |
| Drunkenness | Drunk | Don't get drunk/don't binge drink |
|  | Excess | Don't drink too much/don't drink to excess/in excess/excessively |
| Think | Smart | Be smart, drink smart |
|  | Sensible/safe | Be sensible/make safe choices/have a plan/control the amount you drink |
|  | Minimisation | Pace your consumption/pacing/drink less/drink water in between |
|  | Careful | Be careful |
| Know | Know | Know what you drink |
|  | Stop | Know when to stop |
|  | Limits | Know your limits |
|  | Enjoy | Enjoy but within limits |
| Abstain | N/A | Don't drink alcohol/don't drink any |
| Drive | N/A | Don't drink and drive |
| Increase | N/A | Drink hard/drink more |
| Outcomes | Affect others | Think about everyone who you're affecting when you drink |
|  | Danger | Too much drinking can ruin your life |
|  | Embarrassment | Don’t embarrass yourself |
| Advertisement* | Alcohol | Drink Budweiser when you go out with your friends |
| Sophisticated* | N/A | Be classy, sophisticated |
| Friendship* | N/A | Friends/pets are waiting |

* Code not evident in Study One
