# Supplementary material for: Should I drink responsibly, safely or properly? Confusing messages about reducing alcohol-related harm
Source: PLoS One. 2017 Sep 21;12(9):e0184705. doi: 10.1371/journal.pone.0184705 (PMC5608266; doi:10.1371/journal.pone.0184705)
Supplement: S5 Appendix — (DOCX) [file pone.0184705.s005.docx]

1. **What do the following statements mean to you?**

“Drink Smart”

“You won’t miss a moment if you DrinkWise”

“Drinking: Do it properly”

“Kids absorb your drinking”

“Kids and alcohol don’t mix”

“Know when to say when”

1. **When you hear the phrase ‘kids and alcohol don’t mix’ what age group(s) do you think the word ‘kids’ refers to?**

*(select all that apply)*

- Children aged 0-6 years
- Children aged 7-12 years
- Children aged 13-15 years
- Children aged 16-17 years

1. **What do you think the slogan means in this picture?**

*(select all that apply)*

**
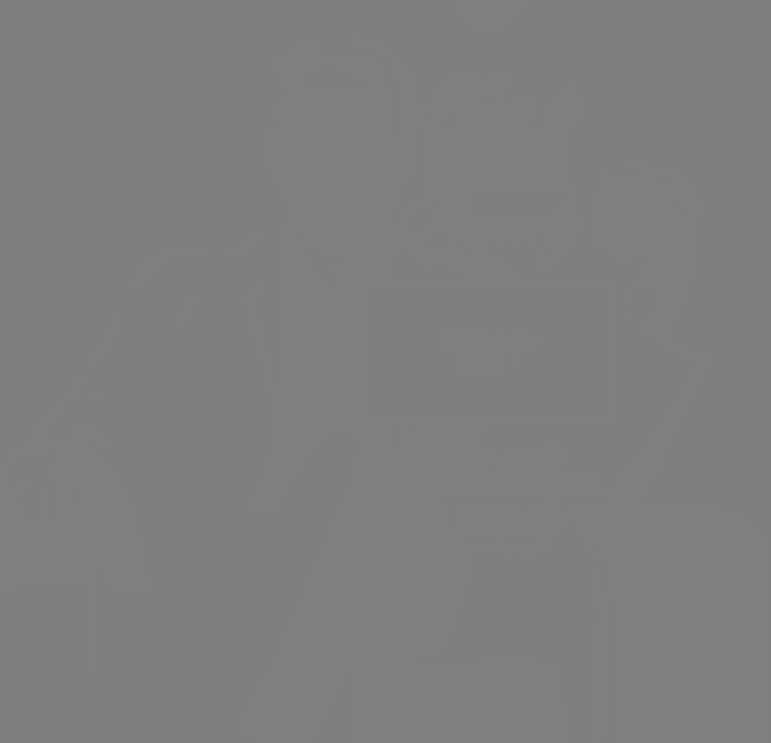
**

- ‘Drinking properly’ means staying sober
- ‘Drinking properly’ means looking cool when you drink
- ‘Drinking properly’ means knowing how to handle your alcohol
- ‘Drinking properly’ means drinking the right kind of alcohol
- ‘Drinking properly’ means knowing your limits
- ‘Drinking properly’ means knowing how to mix a drink
- Other, please specify:

1. **Demographics:** This section of the survey is asking questions about you for statistical purposes.
2. **Are you male or female?**

- Male
- Female
- Other

1. **What is your date of birth?**

_ _ / _ _ / _ _ _ _

DD MM YYYY

1. **What is your present marital status?**

- Married / De facto
- Divorced
- Separated but not divorced
- Widowed
- Never married / single

1. **Are you of Aboriginal or Torres Strait Islander origin?**

- No
- Yes, Aboriginal
- Yes, Torres Strait Islander
- Yes, both Aboriginal and Torres Strait Islander

1. **In which country were you born?**

- Australia
- Other – please specify:__________________

1. **Do you speak a language other than English at home?**

- No, English only
- Yes, other – please specify:_______________

1. **What is your current religion?**

- Anglican (Church of England)
- Catholic
- No religion
- Other – please specify:___________________

1. **What is the highest level of education you have completed?**

- Primary school
- Lower secondary (Year 10 or equivalent)
- Upper secondary (Year 12 / equivalent)
- Certificate or trade / apprenticeship
- Diploma
- Bachelor degree
- Postgraduate qualification or degree

1. **What is the total of all household wages/salaries, government benefits, pensions, allowances and other income you usually receive before tax?**

- Nil / no income
- $1 - $15,599 per year
- $15,600 - $31,199 per year
- $31,200 - $51,999 per year
- $52,000 - $77,999 per year
- $78,000 or more per year

1. **What is your current employment status**

*(select all that apply)*

- Full-time
- Part-time
- Casual
- Looking for work
- Not in the workforce
- Student

*This is the end of the survey, thank you for your participation!*
