# Supplementary material for: Should I drink responsibly, safely or properly? Confusing messages about reducing alcohol-related harm
Source: PLoS One. 2017 Sep 21;12(9):e0184705. doi: 10.1371/journal.pone.0184705 (PMC5608266; doi:10.1371/journal.pone.0184705)
Supplement: S6 Appendix — (RTF) [file pone.0184705.s006.rtf]

1. Participant Information


PROJECT TITLE:
ALCOHOL RESPONSIBILITY STATEMENTS


PRINCIPAL  INVESTIGATOR:
PROF. SANDRA C. JONES; Sandra.Jones@acu.edu.au Dear Participant,
You are invited to participate in the research project described below.


What is the project about?
The research project investigates the way that the public responds to alcohol responsibility messages.


Who is undertaking the project?
This project is being conducted by Professor Sandra C. Jones, Director, Centre for Health and Social Research (CHaSR) at the Australian Catholic University (ACU); Professor Simone Pettigrew, Faculty of Health Sciences at Curtin University; and, Professor Kypros Kypri, Senior Brawn Research Fellow, School of Medicine and Public Health at the University of Newcastle.


Are there any risks associated with participating in this project?
Apart from the time for survey completion, we can foresee no risks for you. If you have any questions or concerns related to the survey, you will be given follow­up contact information.


What will I be asked to do and how much time will the project take?
You are being asked to fill out a 15 minute online survey, which asks for your reactions to four alcohol responsibility media campaigns.


What are the benefits of the research project?
You will not have any direct benefit from taking part except for having a chance to contribute to research which will inform advocacy around the need for governments to take a role in the development and dissemination of responsible drinking messages.


Can I withdraw from the study?
Participation in this study is completely voluntary. You are not under any obligation to participate. If you agree to participate, you can withdraw from the study at any time without adverse consequences. Refusal to participate in the study will not affect your relationship with Australian Catholic University.


Will anyone else know the results of the project?
The study results will be published in academic journals related to alcohol responsibility and health promotion. All data will be non­identifiable, published only as aggregate data, and locked in a filing cabinet that only the researchers have access to. Data will be destroyed within accordance with the ACU ethics policies. General study results will be made available to you should you indicate that you wish to receive them.


Who do I contact if I have questions about the project?
For enquiries about the research, you can contact the researchers.


What if I have a complaint or any concerns?
The study has been approved by the HREC at Australian Catholic University (approval number 2014 292V). If you have any complaints or concerns about the conduct of the project, you may write to:


Chair, HREC
c/o Office of the Deputy Vice Chancellor (Research) Australian Catholic University
Melbourne Campus Locked Bag 4115
FITZROY, VIC, 3065
Ph: +61 3 9953 3150
Fax: +61 3 9953 3315
Email: res.ethics@acu.edu.au


Any complaint or concern will be treated in confidence and fully investigated. You will be informed of the outcome. Yours sincerely,
PRINCIPAL INVESTIGATOR: PROF. SANDRA C. JONES


I want to participate! How do I sign up?
In order to participate, you will need to answer the questions, below, and continue to complete the online survey.


*1. I have been provided with information about this project and agree to participate in this research. I understand that I can stop the survey and withdraw at any time. My participation is completely voluntary.
mlj Yes

mlj No

*2. Which of the following age categories do you fit into?
mlj Under 16 years of age

mlj 16­25 years of age mlj 26­45 years of age mlj 46+ years of age
*3. In which state of Australia do you currently reside in?
mlj New South Wales

mlj Victoria

mlj Western Australia

mlj Other

mlj I do not reside in Australia


Click next to start the survey.	
2.	
	

Please take a moment to watch this video and answer the questions that follow.


*4. What do you think is the main message of this ad?
5

6

*5. Which audience do you believe the ad is intended for?
(select all that apply)

Teenagers aged 12­17 years Young adults aged 18­25 years Adults aged 25 +
Parents


*6. Thinking again about the ad you just watched, what does “how to drink properly” mean to you?
5

6

*7. Is this ad relevant to you?
(select yes or no)


mlj
Yes


mlj No

(please specify why you indicated yes or no):

5
6


*8. Would this ad cause you to change your behaviour in any way?
(please explain)
5


6


Click next to continue the survey.	
3.	


Please take a moment to watch this video and answer the questions that follow.


*9. What do you think is the main message of this ad?
5


6

*10. Which audience do you believe the ad is intended for?
(select all that apply)

Teenagers aged 12­17 years Young adults aged 18­25 years Adults aged 25 +
Parents


*11. Thinking again about the ad you just watched, what does “kids absorb your drinking” mean to you?
5


6	


*12. Is this ad relevant to you?
(select yes or no)

mlj Yes

mlj No

(please specify why you indicated yes or no):

5
6

*13. Would this ad cause you to change your behaviour in any way?
(please explain)
5


6


Click next to continue the survey.	
4.	


Please take a moment to watch this video and answer the questions that follow.


*14. What do you think is the main message of this ad?
5


6	


*15. Which audience do you believe the ad is intended for?
(select all that apply)

Teenagers aged 12­17 years Young adults aged 18­25 years Adults aged 25 +
Parents


*16. Thinking again about the ad you just watched, what does “friends are waiting” mean to you?
5


6

*17. Is this ad relevant to you?
(select yes or no)

mlj Yes

mlj No

(please specify why you indicated yes or no):

5
6

*18. Would this ad cause you to change your behaviour in any way?
(please explain)
5


6


Click next to continue the survey.	
5.	
	

Please take a moment to watch this video and answer the questions that follow.


*19. What do you think is the main message of this ad?
5

6

*20. Which audience do you believe the ad is intended for?
(select all that apply)

Teenagers aged 12­17 years Young adults aged 18­25 years Adults aged 25 +
Parents


*21. Thinking again about the ad you just watched, what does “know when to say when” mean to you?
5

6

*22. Is this ad relevant to you?
(select yes or no)


mlj
Yes


mlj No

(please specify why you indicated yes or no):

5
6


*23. Would this ad cause you to change your behaviour in any way?
(please explain)
5


6


Click next to continue the survey.	
6.	


This section of the survey is asking questions about you for statistical purposes.

*24. Are you male or female?
mlj Male

mlj Female

mlj Other


25. What is your date of birth?
DD	MM	YYYY

Birthday	/	/

*26. What is your present marital status?
mlj Married / de facto

mlj Divorced

mlj Separated but not divorced

mlj Widowed

mlj Never married / single

*27. Are you of Aboriginal or Torres Strait Islander origin?
mlj No

mlj Yes, Aboriginal

mlj Yes, Torres Strait Islander

mlj Yes, both Aboriginal and Torres Strait Islander

*28. In which country were you born?
mlj Australia


mlj Other ­ please specify:
5
6	

*29. Do you speak a language other than English at home?


mlj

No, English only


mlj
Yes, other ­ please specify:

5
6


30. What is your current religion?


mlj

Anglican (Church of England)


mlj
Catholic


mlj
No religion


mlj
Other ­ please specify:

5
6


*31. What is the highest level of education you completed?


mlj

Primary school


mlj
Lower secondary (Year 10 or equivalent)


mlj
Upper secondary (Year 12 or equivalent)


mlj
Certificate or trade/apprenticeship


mlj
Diploma


mlj
Bachelor degree


mlj
Postgraduate qualification or degree


32. What is the total of all household wages/salaries, government benefits, pensions, allowances and other income you usually receive before tax?

mlj

Nil / no income


mlj
$1 ­ $15,599 per year


mlj
$15,600 ­ $31,199 per year


mlj
$31,200 ­ $51,999 per year


mlj
$52,000 ­ $77,999 per year


mlj
$78,000 or more per year


*33. What is your current employment status
(select all that apply)
Full­time Part­time Casual
Looking for work


Not in the workforce Student

Click next to continue the survey.	
7.	


This is the end of the survey, thank you for taking part in the research study: Alcohol Responsibility Statements.


You were involved in filling out an online survey which asked about your attitudes related to alcohol responsibility statements. This study is being conducted by Australian Catholic University, Curtin University, and the University of Newcastle to gain a better understanding of alcohol   responsibility statements and their associated media campaigns. The research will inform advocacy around the need for governments to take a role in the development and dissemination of responsible drinking messages.


If you have any personal concerns as a result from participating in this study please contact Chair, HREC at: res.ethics@acu.edu.au


The following are useful resources for information about alcohol responsibility and campaigns funded by the alcohol industry:
•	https://www.mja.com.au/journal/2006/185/11/assessing­wisdom­funding­drinkwise
•	https://www.mja.com.au/journal/2009/190/12/health­experts­reject­industry­backed­funding­alcohol­research


If you would like to receive a summary of the research results please email Professor Sandra Jones at Sandra.Jones@acu.edu.au.
*34. You are eligible to enter a draw to win a $100 iTunes gift voucher. To participate please tick yes below and click next.
mlj Yes

mlj No	
8.	
*35. Are you 18 years of age and older?
mlj Yes

mlj No


Click next to continue.	
9.	
	


You will be asked to provide your name and an e­mail address. E­mail addresses will not be linked to your survey responses. Please click here to enter the draw for 1, $100 iTunes gift certificate.	
10.	


Your parent or guardian may provide their name and e­mail address. E­mail addresses will not be linked to your survey responses. If you wish to enter the draw, please ask your parent or guardian to click here to enter the draw for 1, $100 iTunes gift certificate.	
